# Nurse-Task Matching Decision Support System Based on FSPC-HEART Method to Prevent Human Errors for Sustainable Healthcare

**DOI:** 10.1007/s44196-023-00224-7

**Published:** 2023-04-10

**Authors:** Salih Cihan Koseoglu, Elif Kılıc Delice, Babek Erdebilli

**Affiliations:** 1 Vocational School, Erzincan Binali Yıldırım University, 24002 Erzincan, Turkey; 2 Department of Industrial Engineering, Ataturk University, 25240 Erzurum, Turkey; 3 Department of Industrial Engineering, AYBU University, 06010 Ankara, Turkey

**Keywords:** Human error nurses, FSWARA, PCA, HEART, Decision support system

## Abstract

To increase the levels of sustainability of service quality as well as to ensure satisfaction and assurance of patients in the health sector, minimizing the probability of making mistakes nurses is of great importance. The extent of this probability is considerably affected by task types, physical conditions of the working environment, workload, and working conditions. Moreover, the physical and mental characteristics of nurses also have a colossal influence on this probability. It is also possible to increase the sustainability of health services by matching nurses appropriately to a specific task according to related risk levels, and by balancing their workload accordingly. This study proposes FSPC-HEART method in that purpose, as a new type of human error reduction and assessment technique (HEART) application based on fuzzy step-wise weight assessment ratio analysis and principal component analysis methods. Unlike the methods in the literature, this new method offers a person-specific proactive error prevention approach. With FSPC-HEART, the probability of each nurse to make a mistake, that is, the human error probability (HEP) values are calculated separately for each task. Also, the combined effect of physical and mental workload factors for each employee was taken into account. In the proposed method, the effect of the subjective judgments of the decision-makers on the objectively obtained HEP values was tried to be reduced. The developed nurse-task matching decision support system enables the FSPC-HEART method to be easily used by decision-makers, and to assign employees to tasks with low error probabilities.

## Introduction

Although recent technological advancements, the quality of health care is still dependent on the individual performances of health professionals. It is recognised that workload, working environment, and processes within the scope of the health sector affect the performance of health workers, and that job-related accidents and injuries are unavoidable as a result of the mistakes made by health professionals while delivering health services. Certain psychological, physiological, and environmental factors contribute to the occurrence of these errors. There are numerous elements that influence the likelihood of human error, including forgetfulness, distraction, disrespect for safety, slacking off, weariness, rapid work, inadequate training, physical limitations, and diseases. In addition, mental workload may result in burnout syndrome, panic attacks, depressive personality disorders, injury, death, financial loss, and paralysis. When workload is considered, it becomes apparent that both mental and physical workload are among the leading causes of occupational deformation. To secure the continuation of high-quality health services, it is crucial to avoid such unfavorable scenarios. In life-threatening situations, human errors committed by medical staff are very likely to result in death or malpractice (wrong treatment) with irreparable consequences [1].

Human error is the failure of a planned action to attain the desired outcome. Human reliability is the probability that personnel will complete their tasks in the system within a specified time without making an error. Human reliability analysis (HRA) method, on the other hand, assumes that failure is a possibility during task performance. Such evaluations may assist authorities in taking the required precautions to decrease the possibility of system mistakes, hence enhancing system safety. HRA serves three fundamental purposes: error characterization, error analysis, and error minimization. Human error probability (HEP) is determined using HRA techniques such as human error reduction and assessment technique (HEART), technique for human error rate prediction (THERP), and Bayesian network [2, 3]. The HEART method is utilized to generate HEP values based on error producing conditions (EPCs) and generic task type (GTT) values obtained for particular jobs. The estimated HEP values are utilized by decision-makers in numerous complex system processes [4].

When computing GTT, EPCs, and assessed proportion of affect (APOA) [5], the HEART relies heavily on expert assessment. The collected findings are analyzed using limited methodologies, and subjective outcomes are obtained based on the opinions of experts, which necessarily increases the amount of inaccuracies [6]. Since the value of HEP depends on the subjective evaluations of GTT, EPCs, and APOA, it is a subjective value that may fluctuate depending on which experts conduct the evaluations. The accuracy of the HEP value is mainly dependent on the knowledge and expertise of specialists. Experts are asked to assign a value between 0 and 1 without the use of a predefined evaluation scale for assessing the APOA rating. In general, APOA refers to the opinion of experts regarding the effect of EPCs on the mistake probability for a specific task [7]. Therefore, APOA value is computed subjectively and is highly susceptible to being wrongly calculated. In addition, it influences the work performance, the probability of human error, and the physical and mental workload elements in a negative or positive manner. Evaluation of this circumstance is difficult and fraught with uncertainty. Mental and physical workload elements must be assessed based on task content, types of mental and physical exertion, and the working environment. Also, the probability of human mistake should be investigated by taking the aforementioned aspects into account.

It presents a novel HEART (FSPC-HEART) as a tailored proactive human error prevention method that is based on fuzzy step-wise weight assessment ratio analysis (FSWARA) and principal component analysis (PCA). The APOA values in this proposed method were determined by combining the FSWARA and PCA techniques. To the best of our knowledge, FSWARA and PCA are combined for the first time in the assessment of human error. Using the FSWARA technique, the effect of decision- makers was minimized, and EPC weights were customized for each individual. The FSWARA approach, an analytical method, was used to calculate the important weights of EPCs based on the imprecise subjective assessments of the decision makers, while the PCA method was utilized to quantify the combined influence of physical and mental workload variables for each individual. The PCA approach was used to combine the variables that had various measurement parameters, and the combined factor was then included in HEART as a single factor. As a consequence of this, the values of APOA were ascertained in an objective manner, and the impacts of the decision-makers' subjective judgements on the HEP value were reduced to a minimum. The HEP value was obtained by combining the values of EPCs and GTTs.

The FSWARA method is recognized as an MCDM operation method. In other words, there is no requirement for the number of decision-makers in survey applications, because this method is not included in the classification of multivariate statistical methods. Additionally, incomplete, unavailable, or uncertain information makes it harder to make decisions with assurance. The development of fuzzy MCDM methods was necessitated by the inability of conventional MCDM techniques to successfully address the challenges associated with such imprecise data. The FSWARA approach enables the evaluation process, which gets more complex due to the problems and elements involved in reaching a decision, to be conducted more effectively and realistically, and permits decision-makers to establish their own priorities. This strategy allows evaluators with a concern for the environment and the economy to determine their own priorities [8, 9]. They reported that the importance of evaluators in FSWARA was more than in other techniques.

The proposed FSPC-HEART method takes into account the impact of physical and mental workload elements, as well as their interplay, on the chance of human error. Physical and mental stresses differ from one to individual. Consequently, they have varying impacts on individuals, and task-specific HEP values vary correspondingly. In this regard, the study used the PCA method to identify the impacts of integrated workload that varies by individual. This strategy minimizes the data volume of variables with varying parameters and data kinds, so that they are centered on the origin. Thus, it provides an easier-to-understand graphical data structure by allowing researchers to determine the direction and cumulative impacts of data [10]. The PCA method utilized physical measures as exogenous factors and mental measurements as endogenous variables. Moreover, correlation and covariance correlations between these variables were considered [11].

As a result of the COVID-19 pandemic and the need to make measurements from a particular distance without interrupting active operations, the present study considers temperature variation, sleep pattern, and age as physical variables. The mental components evaluated by NASA Task Load Index (NASA-TLX) were considered to be the study's mental factors. Individuals may feel more weary based on the duration and volume of their current workload as well as their physical strength [12]. Mental workload can be defined as the interaction between task requirements, environmental conditions, skill and experience levels, behaviors and perceptions, as well as the alterations in mental exertion based on a person's current vitals. NASA-TLX analyzes these alterations based on six-to-nine parameters [13].

Using the FSPC-HEART approach, the HEP values for each individual and each task are objectively calculated, and the tasks that individuals should not be assigned are decided accordingly. Thus, decision-makers are supplied with a proactive approach to error prevention. The FSPC-HEART technique provides a variety of recommendations for minimizing ergonomy-related risk to achieve simple and objective results. The reason for applying the proposed FSPC-HEART approach for nurses in the present study is that they work in an industry characterized by a high risk of human mistake and the importance of human reliability. Providing effective and dependable nursing care, communicating effectively both verbally and nonverbally, and providing both acute and routine help are relatively common daily tasks for nurses. Inadequate staffing, mobbing, and complaints from disgruntled patients increase workload, which in turn severely impacts the mental health of health personnel [14].

In light of increased physical and mental stresses owing to COVID-19, the present study sought to determine the HEP values of nurses for each activity. Thus, based on their HEP levels, the tasks that nurses should not perform were defined.

Because of a lack of theoretical expertise on the subject, the adoption of the FSPC-HEART approach by hospital administration or approved medical staff will be extremely challenging. In addition, the method's implementation will need time and incur expenses. Due to these factors, a user-friendly decision support system (DDS) based on the FSPC-HEART method has been developed for healthcare workers and hospital administration. With this DSS, which is known as the nurse-task matching decision support system (NTM-DSS), the likelihood that a nurse will make an error will be determined, and nurse-task matching will be facilitated based on these probabilities. The sustainability of health services will be assured by assigning registered nurses to positions with a lower error rate. The FSPC-HEART method is a proactive approach to error prevention. Using this method to build the NTM-DSS provides for the prevention of occupational accidents and errors by not assigning nurses over the allowable error probability limits. Consequently, the viability of high-quality health services can be ensured.

**Fig. 1:**
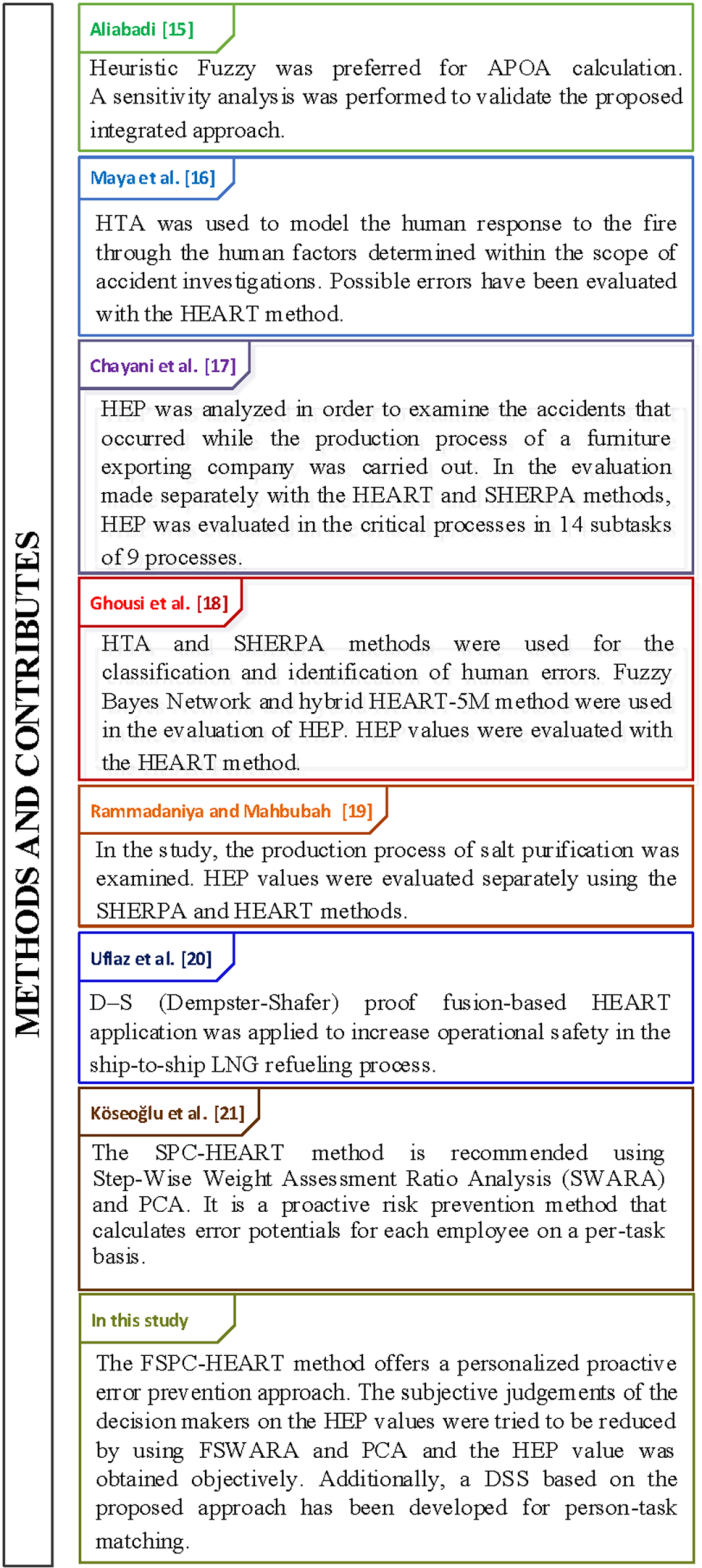
Literature review for HEART method

In the literature review, it was revealed that the number of research completed with the HEART approach was low prior to 2020, but has increased since then. Current literature articles and the contributions of this study to the literature are presented in Fig. 1.

There is limited research on error prevention and the application of the HEART approach in the health industry. The present study aims to contribute to comparable future research by examining the error-making potentials of nurses using a real-world application. In addition, a new DSS has been created to aid hospital administration in decision-making and contribute to HRA research in these other areas.

The following section of the paper provides an explanation of the study's methodology, physical and mental workload components, the proposed FSPC-HEART method, and NTM-DSS. In the third part, the application for nurses is explained. The conclusion and suggestions are presented in the concluding section.

## Materials and Methods

In this section, information about the HEART, FSWARA, and PCA methods and the variables used to calculate the HEP value will be presented. The symbols used in the study and their definitions are summarized in Table 1.

**Table 1:** Nomenclature

Symbol	Definitions	Symbol	Definitions
${\mathrm{EPC}}_{j}$	*j*th error promoting condition (*j* = 1, 2, 3, …, *n*)	${\mathrm{eig}}_{i}$	*i*th eigenvector
${\mathrm{APOA}}_{\mathrm{j}}$	Expert's assessment of the proportion effect for each *j*th EPC	${({e}_{i,j })}^{T}$	Transpose of ${e}_{i,j}$ matrix
${\widetilde{s}}_{j}$	Scientific EPC for evaluation of the comparative significance of average value based on the list of attributes	$\lambda$	Eigenvalue
${\widetilde{k}}_{j}$	The value of coefficient for *j*th factor	${\rm I}$	Unit vector
$\widetilde{{q}_{j}}$	Recalculated weight for *j*th factor	$\Sigma x$	An eigenvector which’s been associated with an eigenvalue
$\widetilde{{w}_{j}}$	The relative weight value of *j*th factor	${W}_{{i}_{f}}$	Weighted value of the *f*th factor level of *i*th participant
$\widetilde{X}$	Triangular number	${\mathrm{WWL}}_{i}$	WWL for *i*th participant
$d\left(\widetilde{X}\right)$	Defuzzification of the fuzzy relative importance weights of the factors	$\Delta {\overline{T}}_{{i}_{b}}$	Body temperature change for *i*th participant
${\widetilde{k}}_{j}^{l}$	The value of coefficient for *j*th factor of lowest point of triangle	$\Delta {T}_{{i}_{c}}$	Core temperature change for *i*th participant
${\widetilde{k}}_{j}^{m}$	The value of coefficient for *j*th factor of middle point of triangle	$\Delta {T}_{{i}_{sk}}$	Shell(skin) temperature change for *i*th participant
${\widetilde{k}}_{j}^{u}$	The value of coefficient for *j*th factor of upper point of triangle	$X$	Alpha (*X*) value
${\widetilde{q}}_{j}^{l}$	Recalculated weight for *j*th factor of lowest point of triangle	${{wp}_{i}}_{m}$	Weighted impact ratio of menthal workload for *i*th participant
${\widetilde{q}}_{j}^{m}$	Recalculated weight for *j*th factor of middle point of triangle	${{wp}_{i}}_{a}$	Weighted impact ratio of age for *i*th participant
${\widetilde{q}}_{j}^{u}$	Recalculated weight for *j*th factor of upper point of triangle	${{wp}_{i}}_{sp}$	Weighted impact ratio of sleep phase for *i*th participant
${\widetilde{w}}_{j}^{l}$	The relative weight value of *j*th factor of lowest point of triangle	${{wp}_{i}}_{t}$	Weighted impact ratio of thermoregulation for *i*th participant
${\widetilde{w}}_{j}^{m}$	The relative weight value of *j*th factor of middle point of triangle	${wp}_{i}$	Weighted impact ratio of for *i*th participant
${\widetilde{w}}_{j}^{u}$	The relative weight value of *j*th factor of upper point of triangle	${\mathrm{APOA}}_{{i}_{j}}$	Proportion effect of the *j*th criterion level of *i*th participant
${F}_{m}$	The structure of mental workload change	${\mathrm{HEP}}_{{i}_{t}}$	Human error probability of the *t*th task of *i*th participant
${F}_{p}$	The structure of physical workload change		
${F}_{PC}$	The structure of principal component change		
${x}_{ij}$	Value of the *j*th factor level for *i*th participant		
${X}_{c}$	Mean-subtracted (centered) data matrix		
${e}_{i,j}$	The matrix with the eigenvectors in the columns		

### HEART Method

Developed in 1986, HEART method is a technique employed in the field of HRA for the purpose of evaluating probability of human error during the course of a specific task. The method takes into account all the factors that might negatively affect a task that is believed to be dependent on human reliability, examines each single factor independently, and measures these factors to obtain HEP value. HEART method is a rather straightforward technique used while calculating HEP values that are generally examined in task-based research [3]. It is based on the principle that there is always probability of an error each time when a specific task is performed and this probability is affected by one or more EPCs. A total of 38 EPCs and nine GTTs—i.e., comparison of different task types—were defined in the 1985 version of traditional HEART method [22]. A different EPC weight value is used while calculating HEP values for each task. By doing so, they prove the relationship of GTT with the tasks

1 $$HEP = GEP \times \mathop \prod \limits_{j = 1} \left[ {\left( {EPC_{j} - 1} \right)APOA_{j} + 1} \right],$$

where generic error probability (GEP) is the error probability value of relevant GTT which is determined by experts. ${\mathrm{EPC}}_{j}$ is based on expert opinion and ${\mathrm{EPC}}_{j}$ is the *j*th (*j* = 1, 2, 3, …, 38). ${\mathrm{APOA}}_{j}$ (from 0 to 1) is termed as the importance of each EPC [3].

### FSWARA Method

Developed by [23], FSWARA is an MCDM technique that follows the steps explained below while determining criteria weights:

*Step 1* Determining importance rankings of the factors.

Let us assume that there are “*n*” number of factors (${C}_{j}$, *j* = 1, 2, …, *n*) and “*k*” number of decision-makers (KV_*k*_, *k* = 1, 2, …, *K*) in the decision-making phase. Each decision-maker ranks all the criteria according to order of importance—the most important one being the first in the list—depending on their knowledge and experience. Later, an integrated ranking is obtained by combining the rankings made by decision-makers.

For the determination of the relative importance scores of tangible and intangible criteria, the fuzzy comparison scale presented in Table 2 has been applied.

**Table 2:** Linguistic comparison scale triangular fuzzy values for evaluation criteria [24]

Linguistic comparison scale for importance weights	Triangular fuzzy values for evaluation criteria (*l, m*, *u*)	
	Response scale	Triangular fuzzy number scale
Equally important	(1, 1, 1)	(1, 1, 1)
Moderately less important	(2/3, 1, 3/2)	(0.67, 1, 1.50)
Less important	(2/5, 1/2, 2/3)	(0.40, 0.50, 0.67)
Much less important	(2/7, 1/3, 2/5)	(0.29, 0.33, 0.40)
Quite less important	(2/9, 1/4, 2/7)	(0.22, 0.25, 0.29)

All relative scores of evaluation criteria have been determined, and using the arithmetic means of the corresponding scores, subjective judgements belongs to decision-makers has been aggregated. The name of this ratio is known as comparative importance of average values [25].

*Step 2* Determining relative levels of importance for each factor.

By taking the integrated ranking into consideration, each decision-maker determines the EPC importance weight by comparing *j*th factor with *j* − 1th factor, which has higher level of importance. Then, the relative importance level—i.e., the ${\widetilde{s}}_{j}$ value—is obtained by calculating the mean of the significance weights determined by each decision-maker.

*Step 3* Calculating the ${k}_{j}$ coefficient.

The $k_{j} \user2{ }$ coefficient is calculated for each factor by utilizing Eq. (2) as follows: The most important factor $\tilde{k}_{j} \user2{ }$ coefficient in the common ranking of the factors is assigned as 1:

2 $$\tilde{k}_{j} = \left\{ {\begin{array}{*{20}c} 1 & {j = 1} \\ {\tilde{s}_{j} + 1} & {j > 1} \\ \end{array} } \right.\quad \tilde{k}_{j} = (\tilde{k}_{j}^{l} ,\tilde{k}_{j}^{m} ,\tilde{k}_{j}^{u} ).$$

*Step 4* Calculating ${q}_{j}$ significance vector.

The $\tilde{q}_{j} \user2{ }$ coefficient is calculated for each factor by utilizing Eq. (3) as follows: the most important factor $\tilde{q}_{j} \user2{ }$ coefficient in the common ranking of the factors is assigned as 1:

3 $$\tilde{q}_{j} = \left\{ {\begin{array}{*{20}c} 1 & {j = 1} \\ {\frac{{\tilde{q}_{j} - 1}}{{\tilde{k}_{j} }}} & {j > 1} \\ \end{array} } \right.\quad \tilde{q}_{j} = (\tilde{q}_{j}^{l} ,\tilde{q}_{j}^{m} ,\tilde{q}_{j}^{u} ).$$

*Step 5* Calculating the fuzzy relative importance weights of the factors.

All factor loadings are calculated by utilizing Eq. (4)

4 $$\widetilde{{w_{j} }} = \frac{{q_{j} }}{{\mathop \sum \nolimits_{k}^{n} q_{k} }}\quad j = 1,2,3, \ldots ,n.$$

*Step 6* Defuzzfying the fuzzy relative importance weights of the factors.

The weights expressed with triangular fuzzy numbers are defuzzified using Eq. (5) to obtain the final criterion weights [26]

5 $$w_{j} = \frac{{\left( {\tilde{w}_{j}^{u} - \tilde{w}_{j}^{l} } \right) + \left( {\tilde{w}_{j}^{m} - \tilde{w}_{j}^{l} } \right)}}{3} + \tilde{w}_{j}^{l} .$$

Assuming the values of ${w}_{j}$ to be between 0 and 1

$$\mathop \sum \limits_{J = 1}^{n} w_{j} = 1.$$

### PCA Method

**Fig. 2:**
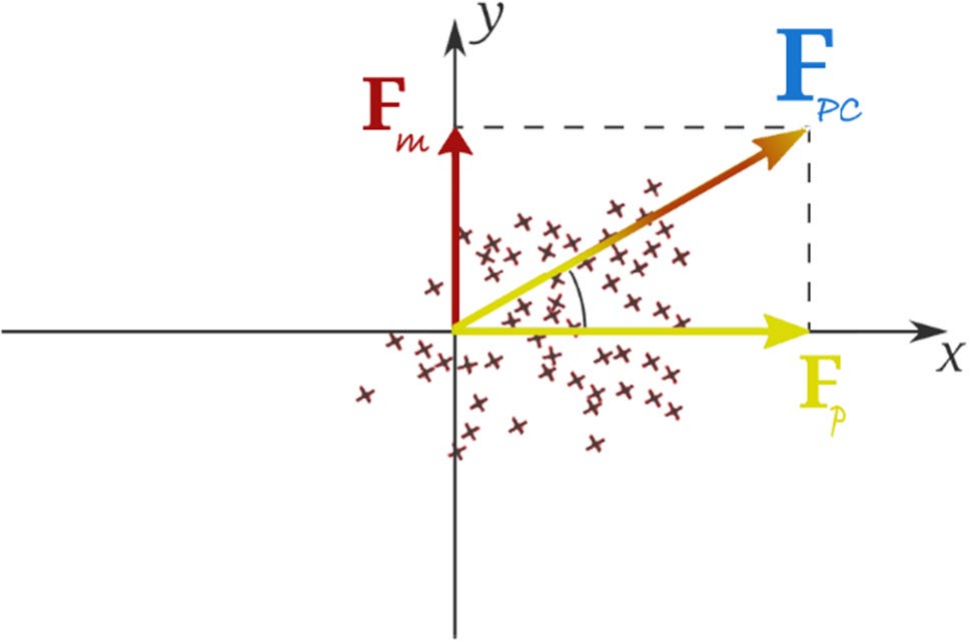
Illustration of mental and physical workload as forces compound [21]

PCA is a multifactor statistical analysis technique employed to explore the structure of the relationship between factors. It basically aims to minimize the size of data set involving a lot of interrelated factors. PCA is based on the idea of transforming correlated factors in a data set into orthogonal factors which do not correlate with each other but have the same number as some linear transformations. These new factors are a linear combination of the existing factors and they are called principal components [27]. If the PCA used in this article is thought like as mechanics, a force can be resolved into its components—two orthogonal forces that are geometrically related to it, as shown in Fig. 2 which describes y axis as mental workload and x axis as physical workload.

In Fig. 2, ${F}_{m}$ shows the structure of mental workload change, ${F}_{p}$ shows the structure of physical workload change, and ${F}_{PC}$ shows the structure of principal component change. PCA generally consists of five basic steps [28].

*Step 1* Raw data standardization and data centralization.

First, raw data are collected for each participant’s measurement factor. If factors do not have the same measurement units, it is necessary to use standardized observation values. Therefore, column mean is calculated for each factor $({x}_{ij})$ and the data are centralized [29]. The centralized data matrix is displayed in Eq. (6)

6 $$X_{c} = \left[ {\begin{array}{*{20}c} {x_{11} - \overline{x}} & \cdots & {x_{1n} - \overline{x}} \\ \vdots & \ddots & \vdots \\ {x_{m1} - \overline{x}} & \cdots & {x_{mn} - \overline{x}} \\ \end{array} } \right]\quad i = 1 \ldots m\;{\text{and}}\;j = 1 \ldots n.$$

*Step 2* Calculating eigenvalues of covariance/correlation matrix and eigenvectors.

Correlation matrix is used when factors have different measurements and all factors are evaluated equally [30]. Covariance matrix is shown in Eq. (7). It is necessary to use determinant calculation using Eq. (8), so that eigenvalue and eigenvectors of covariance matrix can be obtained

7 $${\text{cov}}_{{X_{c} }} = \left[ {\begin{array}{*{20}c} {{\text{cov}} \left( {X,X} \right)} & {{\text{cov }}\left( {X,Y} \right)} \\ {{\text{cov}} \left( {X,Y} \right)} & {{\text{cov}} \left( {Y,Y} \right)} \\ \end{array} } \right],$$

8 $$\Sigma x = \lambda {\rm I}x \Rightarrow \left( {\Sigma - \lambda {\rm I}} \right)x = 0 \Rightarrow \det \left( {\Sigma - \lambda {\rm I}} \right) = \det \left( {\begin{array}{*{20}c} {{\text{cov}} \left( {X,X} \right) - \lambda } & {{\text{cov}} \left( {X,Y} \right)} \\ {{\text{cov}} \left( {X,Y} \right)} & {{\text{cov}} \left( {Y,Y} \right) - \lambda } \\ \end{array} } \right).$$

The squares calculated will give $\left\{{\lambda }_{1},{\lambda }_{2}\right\}$ eigenvalues.

*Step 3* Calculating eigenvalues and eigenvectors of the matrix.

Eigenvalues are ranked descending and the corresponding eigenvectors are calculated using Eqs. (9) and (10) [31]

9 $$\left( {\begin{array}{*{20}c} {\begin{array}{*{20}c} {{\text{cov}} \left( {X,X} \right)} & {{\text{cov}} \left( {X,Y} \right)} \\ {{\text{cov}} \left( {X,Y} \right)} & {{\text{cov}} \left( {Y,Y} \right)} \\ \end{array} } \\ \end{array} } \right)\left( {\begin{array}{*{20}c} {e_{1,j} } \\ {e_{2,j} } \\ \end{array} } \right) = \lambda_{1} \left( {\begin{array}{*{20}c} {e_{1,j} } \\ {e_{2,j} } \\ \end{array} } \right) = \left[ {\begin{array}{*{20}c} {\lambda_{1} e_{1,j} } \\ {\lambda_{1} e_{2,j} } \\ \end{array} } \right],$$

10 $$\left( {\begin{array}{*{20}c} {\begin{array}{*{20}c} {{\text{cov}} \left( {X,X} \right)} & {{\text{cov}} \left( {X,Y} \right)} \\ {{\text{cov}} \left( {X,Y} \right)} & {{\text{cov}} \left( {Y,Y} \right)} \\ \end{array} } \\ \end{array} } \right)\left( {\begin{array}{*{20}c} {e_{i,1} } \\ {e_{i,2} } \\ \end{array} } \right) = \lambda_{2} \left( {\begin{array}{*{20}c} {e_{i,1} } \\ {e_{i,2} } \\ \end{array} } \right) = \left[ {\begin{array}{*{20}c} {\lambda_{2} e_{i,1} } \\ {\lambda_{2} e_{i,2} } \\ \end{array} } \right].$$

*Step 4* Constructing feature vector.

All eigenvectors are assigned as the basic component in an order; the biggest eigen vector being the first basic factor.

*Step 5* Calculating the new data set.

The new data set is obtained by multiplying eigenvectors by the transposed data set. ${e}_{1}\dots {e}_{m}$ values will be new dimension vectors. The new coordinates of the centralized values due to the centralization process will be ${x}^{^{\prime}}=\left\{{{x}^{^{\prime}}}_{1}\dots {{x}^{^{\prime}}}_{m}\right\}$. ${e}_{i,j}$ matrix is the eigenvector matrix where varimax rotation is made. $\left({e}_{i,j }\right)$ is the matrix with the eigenvectors in the columns, as shown in Eq. (11)

11 $$\left( {e_{i,j } } \right) = \left( {{\text{eig}}_{1} {\text{eig}}_{2} {\text{eig}}_{3} \ldots {\text{eig}}_{n} } \right).$$

When = 1 … *m* and *j* = 1 … *n*, the new data set is obtained by multiplying ${e}_{i,j}$ matrix by the transposed ${X}_{c}$ as shown in Eq. (12)

12 $$X_{c} \left( {e_{i,j } } \right)^{T} \quad i = 1 \ldots m\quad j = 1 \ldots n.$$

### NASA-TLX Method

NASA-TLX, which is a subjective workload assessment tool for operators working using human–machine interface, was developed by the Human Performance Group at NASA's Ames Research Center as the outcome of the simulations carried out for 3 years in over 40 different laboratories. It has been proven to be more reliable and more valid than similar mental workload methods. NASA-TLX is a mental workload assessment tool that gives an overall workload score based on the weighted means of 6 or 9 factor scales, since it uses a multi-dimensional scaling procedure. The present study used nine-factor NASA-TLX which involves the following factors: task difficulty (TD), time pressure (TP), performance (P), mental/sensory effort (MSE), physical effort (PE), frustration level(FL), stress level (SL), fatigue (F), and activity type (AT). Stress levels (SL), fatigue (F), and activity type (AT) are the three factors used in the nine-factor NASA-TLX that are different from the 6-factor NASA-TLX. Since these three factors also have an effect in increasing the probability of human error, the study used 9-factor NASA-TLX method. It is possible to measure and assess weighted workload subjectively using these factors.

NASA-TLX method consists of three stages: scaling, weighting, and determining overall workload. In the scaling stage, the effect of nine sub-factors on the tasks is evaluated by marking on a scale ranging between “very low” and “very high”. The values obtained between 0 and 100 after these markings are unweighted workload values [31]. In the second stage called weighting, each participant decides on a weight for each factor according to its contribution to workload. Pairwise Technique (PWT) is used to determine the weights. In this technique, a total of 36 comparisons are made between 9 factors in terms of level of importance according to the work content. Participants mark the criteria which they think contribute to workload the most in these binary comparisons. Later, the frequency value is obtained, i.e., how many times each criterion is selected. In the last stage, ${\mathrm{WWL}}_{i}$ is determined using Eqs. (13)–(14)

13 $$W_{{i_{f} }} = \frac{{{\text{The}}\,{\text{total}}\,{\text{number}}\,{\text{of}}\,{\text{markings}}\,{\text{for}}\,{\text{the}}\,{\text{relevant}}\,{\text{factor }}}}{36},$$

14 $${\text{WWL}}_{i} = {\text{TD}}_{i} xW_{{i_{{{\text{TD}}}} }} + {\text{TP}}_{i} xW_{{i_{{{\text{TP}}}} }} + P_{i} xW_{{i_{P} }} + {\text{MSE}}_{i} xW_{{i_{{{\text{MSE}}}} }} + {\text{PE}}_{i} xW_{{i_{{{\text{PE}}}} }} + {\text{FL}}_{i} xW_{{i_{{{\text{FL}}}} }} + {\text{ SL}}_{i} xW_{{i_{{{\text{SL}}}} }} + F_{i} xW_{{i_{F} }} + {\text{AT}}_{i} xW_{{i_{{{\text{AT}}}} }} .$$

Here, *f* takes TD, TP, *P*, MSE, PE, FL, SL, *F*, and AT values, respectively. ${\mathrm{TD}}_{i}$ is the score value of TD factor for *i*th participant.

### Proposed NTM-DSS-Based FSPC-HEART Method

**Fig. 3:**
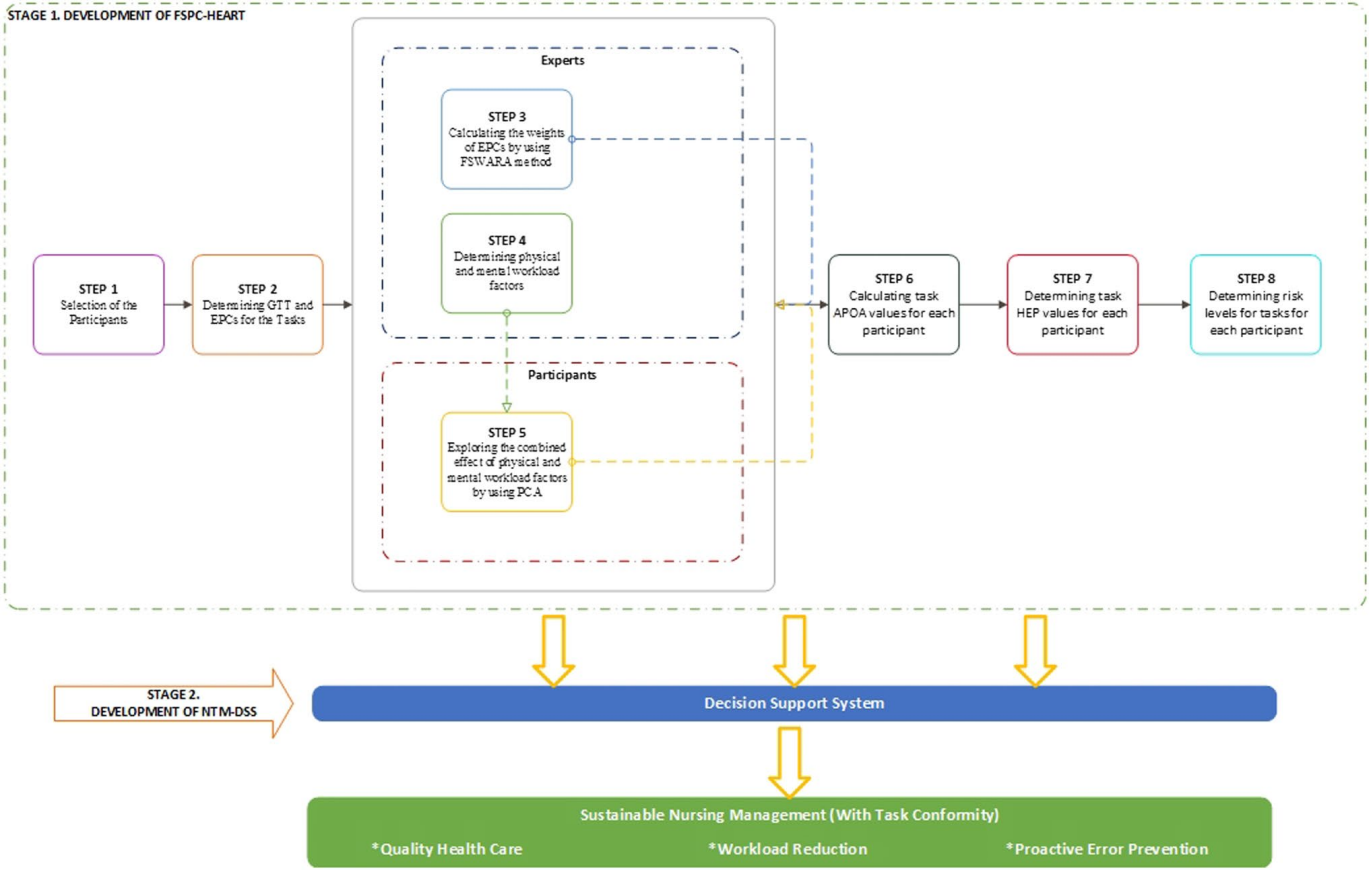
Stages of NTM-DSS-based FSPC-HEART method

To measure task-based HEP, the FSPC-HEART method suggested in the present work presents a new methodology with objective answers that are both experimental and based on expert opinion. Calculating individual-specific HEP values in the FSPC-HEART approach based on quantifiable physical and mental qualities is a substantial improvement over the traditional HEART method. Moreover, analyzing risk factors separately while disregarding their interconnections is likely to have a negative influence on reliability [32]. Each factor's direct and indirect effects have distinct influence on the process. To determine the error tendencies of users in this study, the parameters of the suggested technique were established by incorporating mental and physical strains. Considering that they introduce a variety of concepts and results, assessments based on the combined effects of distinct elements are likely to provide significant insights. Figure 3 presents the flow diagram NTM-DSS-based FSPC-HEART method [33].

In the proposed FSPC-HEART approach, PCA is performed to the input data using Eqs. (6–12), and the weighted composite effects matrix is produced using Eq. (15). The weighted composite effects in this matrix {${wp}_{{i}_{m}}$, ${wp}_{{i}_{a}}$, ${wp}_{{i}_{sp}}$, ${wp}_{{i}_{t}}$}, as indicated in Eq. (16) ${wp}_{i}$ values are then created by combining the weighted composite effects with the geometric mean. Thus, these four factors are reflected as a single composite factor in the APOA computation [21]

15 $$X_{c} \left( {e_{i,j } } \right)^{T} = \left[ {\begin{array}{*{20}l} {\begin{array}{*{20}c} {wp_{1m} } & {wp_{1a} } & {wp_{1sp} } \\ \end{array} } & {wp_{1t} } \\ {\begin{array}{*{20}c} {\begin{array}{*{20}c} {wp_{2m} } \\ {wp_{3m} } \\ {\begin{array}{*{20}c} \vdots \\ {wp_{im} } \\ \end{array} } \\ \end{array} } & {\begin{array}{*{20}c} {wp_{2a} } \\ {wp_{3a} } \\ {\begin{array}{*{20}c} \vdots \\ {wp_{ia} } \\ \end{array} } \\ \end{array} } & {\begin{array}{*{20}c} {wp_{2sp} } \\ {wp_{3sp} } \\ {\begin{array}{*{20}c} \vdots \\ {wp_{isp} } \\ \end{array} } \\ \end{array} } \\ \end{array} } & {\begin{array}{*{20}c} {wp_{2t} } \\ {wp_{3t} } \\ {\begin{array}{*{20}c} \vdots \\ {wp_{it} } \\ \end{array} } \\ \end{array} } \\ \end{array} } \right],$$

16 $$\left[ {\begin{array}{*{20}c} {wp_{1} } \\ {wp_{2} } \\ {wp_{3} } \\ \vdots \\ {wp_{i} } \\ \end{array} } \right] = \left[ {\begin{array}{*{20}c} {\sqrt[4]{{wp_{{1m}} \user2{x}wp_{{1a}} \user2{x}wp_{{1sp}} \user2{x}wp_{{1t}} }}} \\ {\sqrt[4]{{wp_{{2m}} \user2{x}wp_{{2a}} \user2{x}wp_{{2sp}} \user2{x}wp_{{2t}} }}} \\ {\sqrt[4]{{wp_{{3m}} \user2{x}wp_{{3a}} \user2{x}wp_{{3sp}} \user2{x}wp_{{3t}} }}} \\ {\vdots} \\ {\sqrt[4]{{wp_{{im}} \user2{x}wp_{{ia}} \user2{x}wp_{{isp}} \user2{x}wp_{{it}} }}} \\ \end{array}} \right]$$

Then, utilizing the EPC weights from the FSWARA method and the combined effect values obtained from PCA in Eq. (17), *i* for individual *j*, the following is calculated: it is established the effect ratio of ${APOA}_{{\mathrm{i}}_{\mathrm{j}}}$ (0 ≤ ${\mathrm{APOA}}_{{i}_{j}}$≤ 1):

17 $${\text{APOA}}_{{i_{j} }} = wp_{i} \times w_{j} .$$

In the FSPC-HEART technique, the GEP values, EPC values, and ${APOA}_{{i}_{j}}$ values computed according to the GTT categories of the tasks are utilized in Eq. (18) to determine the HEP value of each task individually [21]

18 $${\text{HEP}}_{{i_{t} }} = {\text{GEP}}_{t} \times \mathop \prod \limits_{j = 1} \left[ {\left( {{\text{EPC}}_{j} - 1} \right){\text{APOA}}_{{i_{j} }} + 1} \right].$$

NTM-DSS has been developed using the information obtained from the FSPC-HEART model, so that healthcare personnel can make fast and accurate decisions. There are few studies in the literature using results from MCDM models in a DSS [34, 35].

The NTM-DSS is simulated by merging diverse human-based error reporting modules with varying perspectives. Using the FSPC-HEART approach, HEP values are computed for each person and task, and the risk percentages associated with the tasks are displayed on a user-friendly interface. It was developed using the C# (C-Sharp) programming language for this investigation.

User interface management is used by NTM-DSS to create an interactive user experience. As model management, it offers process alternatives to the user. As data management, it generates both graphical and database outputs. Utilizing OLAP techniques, it analyzes the production process's decision models. This DSS mission is governed by a sustainable personnel management plan; it strives to deliver great customer service, to decrease workload, to share responsibilities, and to help prevent errors.

## Real-Life Application

The purpose of this application is to investigate the effect of nurses' mental and physical exhaustion on their likelihood of making errors, their conduct, and their productivity in the context of risk. The sample size is restricted to daytime employees because of COVID-19 pandemic constraints. The research was conducted without interfering with the nurses' routine work processes, and the nurses' participation throughout the application and its duration had no influence on other procedures. During normal working hours, the researcher administered the survey method and measured the temperature of the nurses' working environments.

After the ethics committee resolution and other legal permits were obtained, the researchers maintained the collected data in digital/hard copy by assigning a code/number/proper name to ensure the confidentiality of the personal information.

*Stage 1* FSPC-HEART method

*Step 1* Selection of the participants

The implementation took place in a Training and Research Hospital. A total of 144 nurses were asked to participate in the study; however, 8 of them refused to be a participant. Accordingly, 136 nurses were determined as the participants of the study: 71.32% female (*N* = 97) and 28.68% male (*N* = 39). 74.41% of these nurses are married (*N* = 74) and 45.59% single (*N* = 62). As for the job experience, 55.88% (*N* = 76) have worked for 5 year and less; 15.44% (*N* = 21) for 6—10 years and 14.71% (*N* = 20) for 10–20 and 13.97% (*N* = 10) for more than 20 years. Also, 92.64% of the participants are service nurses, 6.62% charge nurses and 0.74% (*N* = 1) a head nurse. Finally, 75% (*N* = 102) of the nurses are graduates of an undergraduate program, while 16.91% (*N* = 23) graduated from an associate degree program. The departments where the participants work are presented in Table 3. The Emergency Unit is the department with the highest number of participants.

**Table 3:** The distribution of the departments where the participants work

Department	Frequency	Department	Frequency
Emergency Medicine	35	General Internal Medicine	3
Neurosurgery	3	Infectious diseases	1
Pediatric Surgery	2	Cardiovascular Surgery	5
Child Health and Diseases	15	Cardiology	4
Child Hematology and Oncology	2	Otorhinolaryngology	3
Pediatric cardiology	1	Orthopedics and Traumatology	3
Pediatric Nephrology	1	Palliative care	5
Others (Departments not on the list)	25	Plastic and Reconstructive Surgery	1
Injection	2	Radiology	1
Physical Medicine and Rehabilitation	2	Rheumatology	2
General Surgery	3	Urology	10
Ophthalmology	5	Intensive care unit	2

*Step 2* Determining the tasks, GTT, and EPCs

This stage involved the process of determining the tasks that nurses perform while doing their job. Accordingly, more than 100 tasks defined for nurses working in Turkey and the world were listed and scored in terms of difficulty. The most difficult 45 tasks were determined in collaboration with the participant nurses according to the results of the brainstorming sessions and the Pareto analysis.

Later, a pilot practice was carried out with 27 nurses (19 female and 8 male nurses) working in the emergency units. They were asked to reply the surveys and score the draft tasks according to their experience and knowledge levels. This pilot practice allowed researchers to make necessary revisions and updates regarding 45 tasks and survey documents. 38 of these tasks were found to involve EPCs (Appendix 1). Finally, GTT and EPCs were determined for each particular task.

*Step 3* Calculating the weights of EPCs using FSWARA method

In this stage, the importance weights for each EPC were calculated using Eqs. (2)–(5) in FSWARA method, as shown in Table 4. Initially, the frequencies of EPCs in 45 tasks were used while calculating $\widetilde{{s}_{j}}$ values.

**Table 4:**
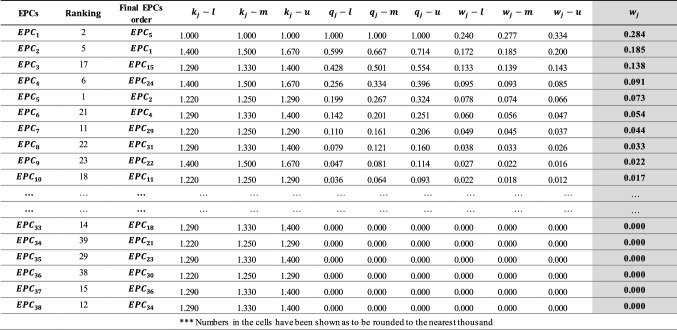
EPC weights calculated with the FSWARA method

*Step 4* Determining physical and mental workload factors

At this stage, physical and mental factors were determined according to task content, types of physical and mental efforts made in the tasks, and working environment. The present study takes temperature change, sleep pattern, and age as the physical factors, since working procedures in the hospitals should not be interrupted and the researchers had to make measurements from a certain distance due to the precautions suggested by the Minister of Health regarding the COVID-19 pandemic. The mental factors used in the study were nine factors measured by NASA-TLX.

**Fig. 4:**
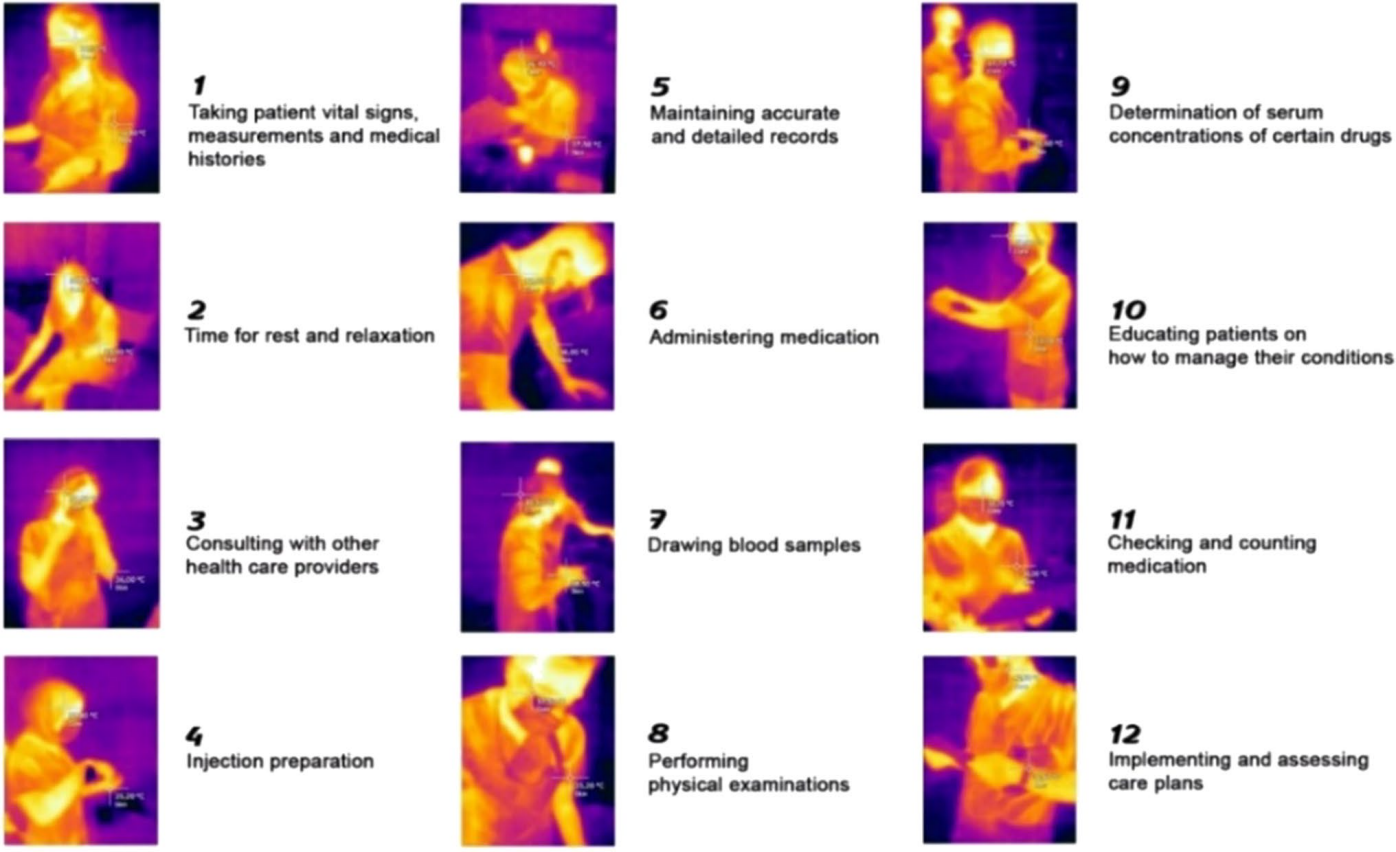
Temperature measurements of nurses

*Temperature change* As for the thermoregulation measurements, the researchers used Infrared Thermography to measure core and shell (skin) temperatures. Thermal infrared cameras were used for thermal temperature measurements from a distance of 50 cm and at 0.98 emissivity measurement value, which is considered a standard value. Figure 4 displays various samples of measurements. The present study used the traditional two-compartment thermometry model suggested by Burton (1935) as in Eq. (19) to identify changes in the average body temperature of the participants [36]

19 $$\Delta \overline{T}_{{i_{b} }} = \left( {X \cdot \Delta T_{{i_{c} }} } \right) + \left( {\left[ {1 - X} \right] \cdot \Delta T_{{i_{sk} }} } \right).$$

Thermoregulation values, which are calculated using Eq. (19) and displayed in Table 5, are within the normal range with the average value of 36.8528.

**Table 5:** Input matrix $({x}_{ij})$ for PCA

Nurses	${\mathrm{WWL}}_{i}$	Thermoregulation	Sleep phases	Age
${N}_{1}$	63.8888	37.33	6.3	23
${N}_{2}$	98.0555	36.35	4.5	24
${N}_{3}$	96.25	36.97	4.5	39
${N}_{4}$	97.9166	36.89	4.5	33
${N}_{5}$	75.4166	36.78	6.3	44
${N}_{6}$	86.1111	37.49	6.3	45
${N}_{7}$	84.3055	36.55	8	32
${N}_{8}$	87.5	36.69	8	45
${N}_{9}$	79.8611	36.96	6.3	25
${N}_{10}$	98.8888	36.83	6.3	29
…	…	…	…	…
…	…	…	…	…
…	…	…	…	…
${N}_{130}$	75	36.84	6.3	22
${N}_{131}$	94.4444	37.81	6.3	30
${N}_{132}$	74.0277	36.42	6.3	33
${N}_{133}$	78.8888	37.33	8	33
${N}_{134}$	89.7222	36.35	8	45
${N}_{135}$	48.8888	36.97	8	25
${N}_{136}$	71.1111	36.89	8	24

*Sleep pattern* Although there is a variety of sleep phases, the present study deals with three sleep phases: single, double, and multiple phase. The single sleep phase refers to an uninterrupted up to 8-h sleep during the night. Double phase sleep means that an individual sleeps up to 6.3 h each time during the night. Finally, multiple phase sleep refers to sleeping for short periods of time within 24 h, which is often preferred by young people. They generally sleep 4.5 h in each phase [37].

*Age* Work performance depends on task-related factors, such as perceived workload, the effects of a working environment, and task content as well as other task-independent factors, such as personal ability, attempts, sensitivity, and age factor [38]. In this respect, it has been reported that elderly employees perceive a considerably higher increase in workload. In addition, a decrease in cognitive performance results in a slower resource processing as the individual gets older, which is also known as cognitive aging (related with multimorbidity) [39]. Also, Salthouse (1991) emphasized age factor as a significant component of cognitive aging [40].

The sleep phases and ages of the nurses were determined using the data collected from the demographic survey and are shown in Table 5. The average duration of standing working was found to be 10.2574 h, and this value implies a significant indicator of exhaustion. In addition, the average for sleep phases was 6.3324 h, which indicates a moderate level.

The nurses were administered a survey while implementing the NASA-TLX method and ${\mathrm{WWL}}_{i}$ displayed in Table 5 was obtained by utilizing the data collected from the survey in Eqs. (13)–(14).

*Step 5* Exploring the integrated effect of physical and mental workload factors using PCA

First, PCA was applied to the input data using Eqs. (6–12) and the weighted composite effect matrix was obtained, as shown in Eq. (15). Then, by applying Eq. (16), the geometric mean and ${wp}_{i}$ value were obtained.

Minitab 18 and IBM SPSS Statistics 26 software were used for the input matrix displayed in Table 5. Table 6 displays the distributions for input weighted data.

**Table 6:** Weighted integrated effects matrix ${{X}_{c}({e}_{i,j })}^{T}$ and ${wp}_{i}$

Nurse	${{wp}_{i}}_{m}$	${{wp}_{i}}_{a}$	${{wp}_{i}}_{sp}$	${{wp}_{i}}_{t}$	${wp}_{i}$
${N}_{1}$	1.443777	0.324434	0.465848	1.615765	0.77057
${N}_{2}$	1.799273	1.183424	0.446812	0.122653	0.584467
${N}_{3}$	1.829892	0.809545	0.214706	0.082996	0.403081
${N}_{4}$	1.852364	0.047155	0.232116	0.014465	0.130864
${N}_{5}$	0.300365	1.684984	0.525727	0.09675	0.400556
${N}_{6}$	0.286286	1.800334	0.789055	0.403092	0.636306
${N}_{7}$	0.761955	0.084774	0.405016	0.960074	0.3981
${N}_{8}$	0.375106	1.405549	0.351812	1.533463	0.730291
${N}_{9}$	0.346477	0.567214	0.188166	0.478263	0.364676
${N}_{10}$	0.940004	0.565001	0.313454	0.846997	0.612786
…	…	…	…	…	…
…	…	…	…	…	…
…	…	…	…	…	…
${N}_{130}$	0.683597	0.849015	0.047585	0.83979	0.390247
${N}_{131}$	0.528612	0.025432	1.731612	0.350309	0.300506
${N}_{132}$	0.508567	0.314833	0.920087	0.438268	0.50408
${N}_{133}$	1.205063	0.410009	0.6806	0.456337	0.625886
${N}_{134}$	0.183853	1.24373	0.835739	1.758451	0.761376
${N}_{135}$	3.175995	0.021988	0.368347	1.539109	0.446066
${N}_{136}$	1.773923	0.612385	0.008552	0.191103	0.20527

*Step 6* Calculating task-based APOA values for each participant

EPC weights obtained at Stage 3 and integrated effect values obtained at Stage 5 are utilized in Eq. (17) to determine ${\mathrm{APOA}}_{{i}_{j}}$. For instance, ${\mathrm{APOA}}_{{14}_{1}}$ value for ${\mathrm{EPC}}_{1}$ for the nurse coded as ${N}_{14}$ is calculated as follows:

$${\text{APOA}}_{{14_{1} }} = wp_{14} \times {\text{w}}_{1} .$$

*Step 7* Determining task-based HEP values for each participant

It is possible to calculate task-based $APO{A}_{{i}_{j}}$ values as well as HEP values for each participant due to different GEP values and EPCs for each task. For instance, the ${HEP}_{{14}_{20}}$ value for the nurse coded as ${N}_{14}$ is calculated using Eq. (18) as follows:

$${\text{HEP}}_{{14_{20} }} = {\text{GEP}}_{20} \times \mathop \prod \limits_{j = 1} \left[ {\left( {{\text{EPC}}_{1} - 1} \right){\text{APOA}}_{{14_{1} }} + 1} \right].$$

Task-specific ${HEP}_{{i}_{t}}$ values calculated for each nurse on are displayed in Table 6.

*Step 8* Determining each participant’s risk levels for tasks

In this stage, the tasks involving risks and the risk-free tasks were determined for the nurses according to ${\mathrm{HEP}}_{{i}_{t}}$ values calculated in the previous stage. The risk categories prepared by Garvey and shown in Table 7 are used to achieve this purpose. ${\mathrm{HEP}}_{{i}_{t}}$ values were categorized according to the risk categories given in Table 7, and the categorized values are shown in Table 8.

**Table 7:** Risk definition ranges [41]

Risk event probability	Interpretation	Rating
> 0 to ≤ 0.05	Extremely sure not to occur	Low
> 0.05 to ≤ 0.15	Almost sure not to occur	Low
> 0.15 to ≤ 0.25	Not likely to occur	Low
> 0.25 to ≤ 0.35	Not very likely to occur	Low
> 0.35 to ≤ 0.45	Somewhat less than an even chance	Medium/moderate
> 0.45 to ≤ 0.55	An even chance to occur	Medium/moderate
> 0.55 to ≤ 0.65	Somewhat greater than an even chance	Medium/moderate
> 0.65 to ≤ 0.75	Likely to occur	High
> 0.75 to ≤ 0.85	Very likely to occur	High
> 0.85 to ≤ 0.95	Almost sure to occur	High
> 0.95 to < 1	Extremely sure to occur	High

**Table 8:**
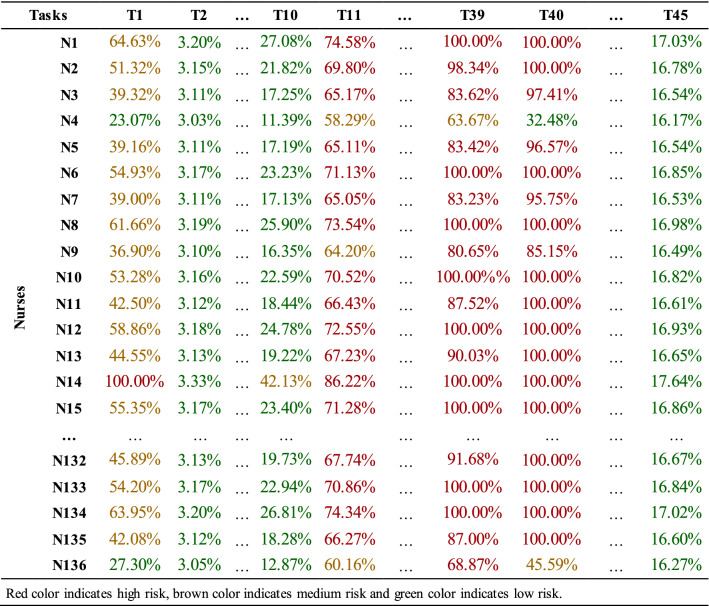
${\mathrm{HEP}}_{{i}_{t}}$ values for nurses

According to Table 8, the values for HEP on an individual basis are quite high due to high EPC values of some tasks. In addition, the tasks that each nurse should never be assigned to depending on their risk levels were determined. For instance, the error probability of the nurse N14 for the tasks T1, T11, T13, T15, T30, T33, T34, T35, T39, T40, and T43 are quite high. In other words, it is assumed that she will make mistakes while performing these tasks. It is a risk to assign this nurse to the following tasks: T1: dealing with emergencies, T11: execution of procedures without the necessary information about the patient's history, T13: conflicts with patients and their relatives, communication disorder, etc. working under conditions such as T15: working within the scope of demands, such as that patient relatives expect nurses to show a certain level of empathy and sensitivity, T30: working in departments where the number of nurses is insufficient in terms of workload, T33: execution of procedures within the framework of negativities, such as incompatibilities, conflicts, etc. with student and trainee nurses, T34: working with lack of time in terms of preliminary preparation and work planning, T35: procedures resulting from lack of practice or encountering for the first time inadequate intervention to critically ill patients, T39: to carry out the procedures within the negativities, such as inability to hear clearly in blood pressure measurement, and incompatibility of the patient's arm and cuff, T40: working under undesirable conditions, such as problems, disorders, etc. in the functioning of medical devices, and T43: execution of transactions with the effects of directives and expectations of superiors.

According to the data obtained, the participant N14 is female service nurse working in the emergency unit. She graduated from an undergraduate program and has less than 5 years of experience. In addition, she has to work standing for 24 h, suffers from sleep disorders, and was diagnosed with mild hypothermia during thermoregulation measurements (34.47 °C).

Moreover, she displays certain symptoms, such as shaking, reduced reasoning, loss of memory, apathy, and increased rate of heart and breathing. The NASA-TLX value for that nurse was calculated as 87.5. Table 9 shows task-risk assessment for the nurse N14, hereinafter.

**Table 9:** Task-risk assessments for N14

Tasks	Rating	Interpretation
T1. T13. T30. T33. T34. T35. T39. T40	High	Extremely sure to occur
T11	High	Almost sure to occur
T15. T43	High	Likely to occur
T31	Medium/moderate	Somewhat greater than an even chance
T10	Medium/moderate	Somewhat less than an even chance
T4. T8. T19. T20. T25	Low	Not very likely to occur
T9. T12. T27. T29. T32. T45	Low	Not likely to occur
T16. T22. T26. T36. T37	Low	Almost sure not to occur
T2. T7. T18. T23. T28. T38. T41. T42. T44	Low	Extremely sure not to occur
Tasks which have no EPCs: T3, T5, T6, T14, T17, T21, T24		

As seen in Table 9, N14 has high-risk values for many tasks. This nurse should receive psychological support to cope with her adaptation problems and she should not be assigned to the tasks with high-risk levels. It is also crucial to take some precautions regarding job safety. In addition, her competence should be improved through in-service training sessions, the job descriptions should be clarified and the scope of work should be narrowed down and specified in written directives, error reports should be prepared in a way to include effective feedback, and internal audits should be made regularly by senior nurses.

*Stage 2* Development of NTM-DSS

The NTM-DSS is simulated by coupling various human-based error reporting modules of different perspectives. Specifically, the human error possibilities are calculated in background, results are generated using FSPC-HEART model, and the corresponding risk possibilities are represented by a user-friendly screen. In this study, NTM-DSS had been built with C# (C-Sharp) programming language.

**Fig. 5:**
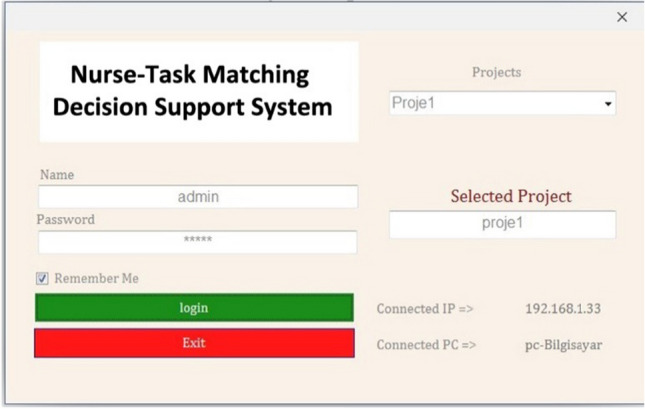
NTM-DSS user interface

First, the user's username and password are requested. At the same time, project selection is presented to the user, as shown in Fig. 5. After making the necessary selections and entering the user information, the system opens.

*Task information definition module* Task definitions are made and the GTT and EPCs of these tasks are determined.

*Demographic data entry module* This is the module that allows the demographic information of the participants to be entered. Data being used encompass a number of occupational info like age, daily sleep quality, sex, title, department, experience, marital status, education, and working hour data belonging to nurses.

*Mental measurement module* It is the module that calculates the NASA-TLX values of the person according to different scales.

*Temperature calculation module* After the core and skin temperature measurements of the participant are entered, the final temperature measurement, which is the thermoregulation value of the person, is calculated.

**Fig. 6:**
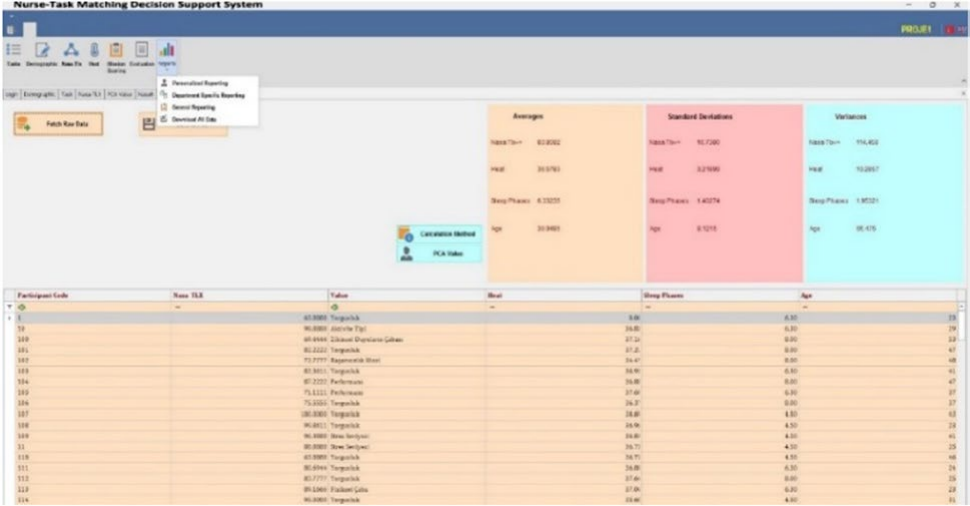
PCA calculation screen

*Calculation module* In this module, first, PCA values are calculated for each participant. While calculating these values, the age, sleep, TLX, and temperature values entered in DSS are used thanks to the previous modules, as shown in Fig. 6.

**Fig. 7:**
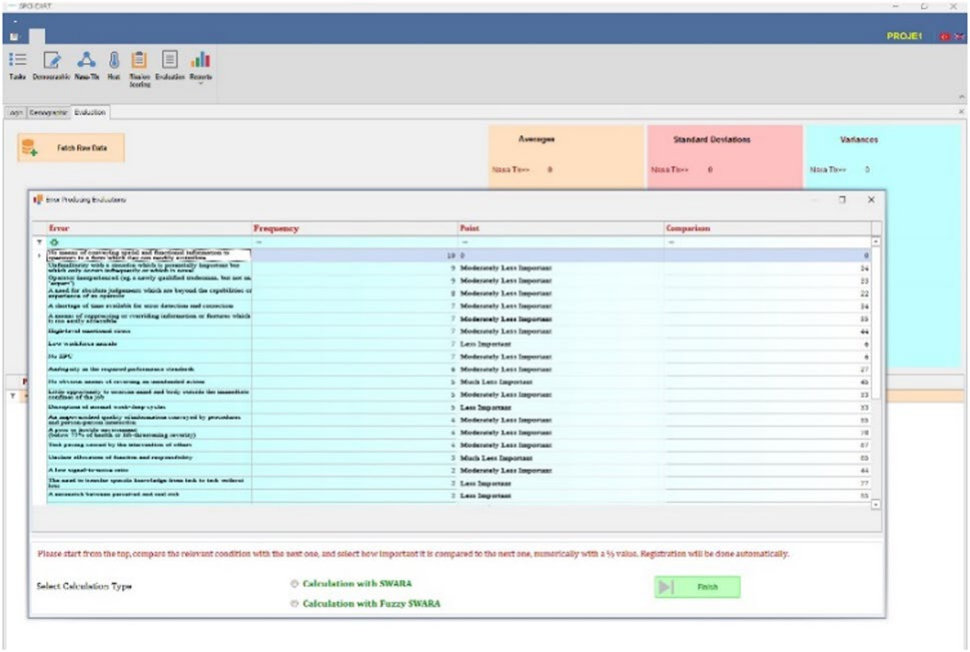
SWARA and FSWARA calculation screen

Second, the importance weights of EPCs are calculated with SWARA or FSWARA, depending on the user's preference (Fig. 7). Thus, the recommended FSPC-HEART or the SWARA version of this model is run in SPC-HEART DSS.

**Fig. 8:**
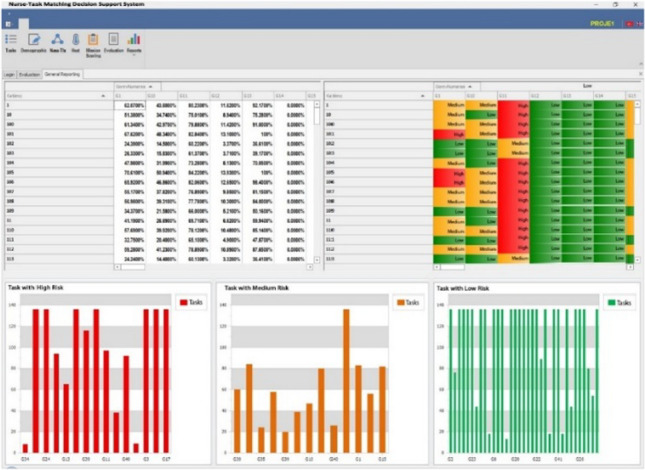
General reporting

*Reporting module* Personal Reporting, Department Reporting, and General Reporting (all participants and all departments) are made. Using these reports, the possibility of nurses to make mistakes on a task basis is revealed and risk assessments are presented to decision-makers. As the risk levels of the tasks are revealed, the decision-makers can easily make the nurse-task assignments considering these risk levels (Fig. 8).

In all three report types, nurses' job risk assessments are shown with numerical tables and graphical windows. There are histogram graphics of high-, medium-, and low-risk tasks in three separate parts at the bottom with the risk assessment table. In these histograms, it can be seen which participant is involved in the relevant task possessed which risk type based on nurse task matching.

## Discussion

### Sensitivity Analysis

In this study, the risk levels of the participants for each task were determined. It was found that thermoregulation, sleeping phases, age, and mental workload as well as task-specific EPCs have considerable effects on these high-risk levels. The distribution of EPCs’ values according to the tasks is presented in Table 10.

**Table 10:** The use of EPCs towards tasks

EPCs	5	1	15	24	2	4	29	31	22	11	7	36	37	16	33	25	3	10	26	28	6	8	9	12	17	19	20	27	32	34	38
NU*	10	9	9	8	7	7	7	7	6	6	5	5	4	4	4	3	2	2	2	2	1	1	1	1	1	1	1	1	1	1	1
Tasks**	13	1	1	4	4	10	7	4	1	2	13	13	4	12	12	33	13	23	35	32	40	23	19	45	40	9	40	41	40	15	33
	19	10	9	8	8	11	10	7	2	7	16	18	22	13	13	35	19	26	40	44											
	20	12	12	10	25	12	11	11	33	22	18	33	33	18	16	44															
	22	15	18	12	30	13	13	13	37	28	37	35	38	33	33																
	26	20	28	27	35	25	29	18	40	44	40	42	40																		
	30	34	33	36	39	35	35	31	42	45																					
	33	35	34	38	40	39	45	35																							
	35	36	40	40																											
	40	40	43																												
	43																														
Tasks with no EPC: 3, 5, 6, 14, 17, 21, 24																															

*NU is the number of total usage for EPCs

**Tasks have been identified with EPCs which were shown above

Table 10 shows that the EPC5 is the most common EPC while performing the tasks. Defined as “no means of conveying spatial and functional information to operators in a form which they can readily assimilate”, the EPC5 should be examined and necessary improvements should be made. To achieve this purpose, it is essential to improve cognitive perception by making use of various communication channels in an integrated way and using audio-visual elements. It is also possible to eliminate EPC5 condition by obtaining optimum productivity through the effective use of technological innovations, such as Google Glass and hybrid data and communication management systems [42], which are the recent developments of Industry 4.0 applications through IoT technologies and explicitly enhances and facilitates the immediate communication between system pillars by designing DSSs with Internet access [43].

The HEP values of the tasks will decrease with the elimination or improvement of the EPCs that are frequently encountered or that have a large impact on the HEP value. For example, T13, T19, T20, T22, T26, T30, T33, T35, T40, and T43 are affected EPC 5. With the elimination of this condition, the new ${\mathrm{HEP}}_{{i}_{t}}$ values calculated for the tasks are given in Table 11 and it is seen that the new ${\mathrm{HEP}}_{{i}_{t}}$ values of the tasks are lower than the old ${\mathrm{HEP}}_{{i}_{t}}$ values. This result is an expected result. For example, if nurses are adequately informed about medical problems and technical malfunctions that may occur in the previous shifts and patient-related emergencies, they will make fewer mistakes while performing their T35 and T40 tasks.

**Table 11:**
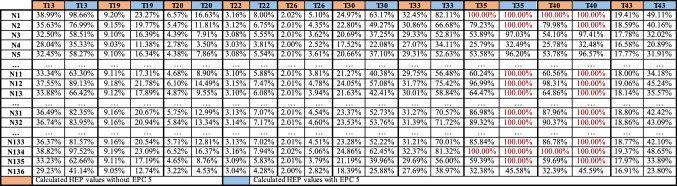
HEP values for tasks with and without EPC5

Different measures should be taken and ergonomic arrangements should be made to reduce the risk levels of tasks with different risk levels. For example, the precautions to be taken according to the risk levels of the tasks for the N14 participant are given in Table 12.

**Table 12:** Suggestive solutions with a view to risky tasks

Propositions	
*High risk*	Providing cognitive and hypnotic treatment support in order to gain operant experience Development of special methods for anamnesis procedures Elimination of negative factors in the working environment Facilitating the orientation processes, developing work compliance methodologies based on reward and punishment Developing procedures to prevent the emotional impact factors of patient relatives
T1, T11, T13, T15, T30, T33, T34, T35, T39, T40, T43	
*Medium risk*	Increasing the number of qualified nurses for reducing the nursing workload Carrying out assessments to increase the motivation of inexperienced nurses Creating simple and explanatory instructions for processes of professional practices
T10, T31	
*Low risk*	Stocking of consumables supply like as in the just-in-time manufacturing strategy Appointment of a sufficient number of nurses in departments Making the work assignments according to experience level Providing fast and continuous stocking opportunities by improving the supply conditions of medical supplies
T2, T4, T7, T8, T9, T12, T16, T18, T19, T20, T22, T23, T25, T26, T27, T28, T29, T32, T38, T41, T42, T36, T37, T44, T45	

As shown in Table 12, with the improvements to be made for each nurse on a task basis, it can be ensured that people make fewer mistakes and thus the risk levels of the tasks can be reduced.

### Comparison Analysis

The outputs of the FSPC-HEART approach are dependent on the variation of the GTT, EPC, and APOA parameters. Changes in APOA or EPC weights have an effect on HEP readings. Table 13 displays the variations in HEP values based on the APOA values determined using the SWARA and FSWARA methodologies.

**Table 13:**
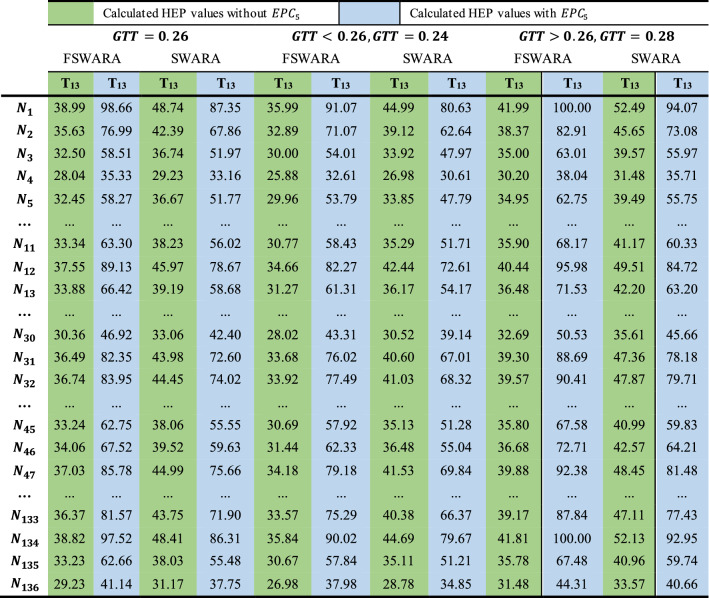
Comparative HEP values for ${\mathrm{EPC}}_{5}$

For the comparison analysis, the standard value of the GTT value, hypothetically lower and higher values for SWARA and FSWARA by clustering separately; when the changes of the G13 task were compared to the changes of the ${\mathrm{EPC}}_{5}$ value in the calculations of ${\mathrm{EPC}}_{j}$ and ${\mathrm{APOA}}_{j}$, the ${\mathrm{EPC}}_{5}$ value was discovered to be significantly effective for all three variables. When comparing the FSWARA and SWARA methodologies, the HEP % values obtained with FSWARA were consistently greater than the SWARA values. As a result of the evaluation process, which became more complex as a result of the difficulties and factors encountered when making decisions in an uncertain environment, the weights were determined more precisely using FSWARA, and realistic HEP values were achieved.

In contrast, the change in terms of GTT followed the same direction as the change in the GTT variable. As the GTT value declined, the resulting HEP values decreased, and as it increased, the acquired HEP values increased.

## Conclusions

In this study, a new approach named FSPC-HEART and a DSS based on this method were developed. Unlike its predecessors, which were simpler and more prevalent, the new method use PCA-constructed centroids to produce more detailed and exhaustive results. It is simpler to obtain more specific APOA values, and it offers a more robust and reliable solution in terms of its application regions. The proposed method, unlike the traditional HEART method, calculates distinct HEP values for each given activity. Each individual's risk value is evaluated, and the jobs they should not be allocated are determined. Individual risk levels can be reduced by planning and arranging preventive activities and requiring individuals to participate in them. Thus, it is possible to achieve the sustainability of health care at a particular quality level. There is little doubt that user-friendly DSSs and technologies will be highly beneficial in increasing healthcare quality and human services.

Within the scope of the current investigation, the risk levels of nurses were established. For some nurses, these levels were very high. Consequently, it may be possible to maintain appropriate levels of stress and mental workload among nurses by obtaining moderate-risk levels for variables like work ethics, ergonomic working environment, team collaboration, team experiences, organization quality, familiarity, and available time. In addition, it is known that all health workers faced emotional and physical stress during the pandemic. It is vital to take additional safeguards, such as increasing the number of nurses, to reduce workload and stressors as a result of the pandemic's unique and hard conditions. All types of enhancements to their working circumstances and settings will increase their productivity.

For consistently superior health services, a continual improvement-development plan requires constant and accurate data sharing. The nurse must have a comprehensive awareness of the treatment history of the patient. The occurrence of these disturbances appears to enhance the chance of nursing errors. Future technological improvements, such as Industry IV or V, fall within the realm of solutions that can be proposed based on study findings. Errors and other occurrences will be avoided by constructing unique systems that provide simultaneous data transmission. Online applications employing artificial intelligence and memory management will achieve the required quality level. In building systems that will benefit from technological advances at both the measurement and management levels, it is anticipated that the risk identification technique presented in this study will serve as the foundation for contemporary evaluation and preventative measures.

Due to the need to ensure the continuity of the business environment and the detrimental effects of pandemic limitations on the implementation process, it was not possible to conduct a comprehensive analysis. This was required to preserve the current workflow and order. In addition, the inability to observe the nurses due to the pandemic outside the working intervals determined 8 a.m. to 4 p.m. and the variability of shifts also negatively affected the work. In spite of the limitations indicated, the obtained data demonstrated that, in addition to the working environment, people's error propensities are also influenced by their individual characteristics. Due to his novel contributions to the existing body of literature, he provided a new perspective on the human–error interaction, which led to the current inquiry.

The architecture of the study makes it possible to conduct new studies in which more variables can be examined in future studies, taking into account the results obtained. The system's capability to monitor EPCs has the potential to serve as the basis for autonomous artificial intelligence systems in future study. It is possible to engage it as a valuable tool at various stages of the institutionalization procedure. In addition, the methods for allocating work and completing tasks will be conducted in a manner that is both healthier and of a higher quality as a result of the potential alterations. EPCs that need to be minimized by developing DSS or EPCs that increase the risk can be presented to the decision-maker by the system and preventive actions can be determined. If new modules are added, updated, or upgraded to the developed DSS, it is possible to use it as an effective solution tool for many future problems. This feature shows the different aspects of NTM-DSS.

In future studies, the DSS developed in this study can be used to determine the risk levels of employees in different sectors. In addition, considering the results of this study, it will be possible to develop a new approach to the personnel assignment problem and to create a solution model by considering different factors, such as cost, experience, working hours, and number of employees, and error minimization. A new HEART method can be developed using different approaches based on uncertainty fuzzy set theory while calculating GTT, EPC, and APOA values. Finally, more comprehensive results can be obtained by including more physical workload and mental workload factors.

## Data Availability

Not applicable.

## Appendix A

### Appendix 1: Selected Nurse Tasks Which Have More Affective on the Nursing Workload

**Table:**

Tasks	
1.	Dealing with emergencies
2.	Working in ambiguous roles or having to carry out conflicting tasks
3.	Directing the patient to the unit by collecting information and completing the admission-transfer procedures
4.	Bringing together more than one job and doing it at the same time
5.	Communication disorders, conflicts, etc. with other team members; working under conditions
6.	Carrying out direct and indirect patient care operations
7.	Carrying out the operations under the mobbing behaviors applied by the teammates
8.	Not having enough information about the operation of the equipment; not being able to respond to the needs in the new conditions, etc., working with obstacles
9.	Simultaneously, the procedures that require physician knowledge are carried out with individual knowledge and experience. since the authorities cannot be reached on time
10.	Performing the procedures for which there is insufficient information about the necessary medication and intervention procedures
11.	Execution of procedures without the necessary information about the patient's history
12.	Not knowing the rights of the patient and carrying out the procedures in this way
13.	Conflicts with patients and their relatives, communication disorder., etc. working under conditions
14.	Inability to communicate effectively with the patient and/or their relatives, etc., executing transactions depending on situations
15.	Working within the scope of demands, such as that patient relatives expect nurses to show a certain level of empathy and sensitivity
16.	Carrying out the current procedures under the pressure of the relatives of the patients to reach the nurse immediately and to meet their needs as soon as possible
17.	Working with the state of burnout caused by the patient/patient relatives' making a choice among nurses and separating them as good–bad and successful–unsuccessful
18.	Working under conditions where patient–nurse trust is not sufficient or cannot be established
19.	Not being able to communicate the changes in vital signs about the patients to the physician in a short time and working within these results
20.	Assisting patients with activities of daily living
21.	Working with the exposure of interacting directly with the suffering experienced by patients
22.	The inadequacy of the relevant department of the hospital in the provision of service tools and equipment. etc. to carry out transactions within the framework of disruptions
23.	Preparation of patient reports
24.	Working simultaneously with the entire healthcare team to support the patient
25.	Measuring and monitoring vital signs; carrying out drug management procedures
26.	Evaluating and planning nursing care needs; carrying out patient follow-up procedures
27.	Finding a lack of data in nursing care and needs, evaluation and planning, and continuation of studies in this way
28.	Working with problems such as not being able to provide necessary materials, such as drugs and serum on time, etc
29.	Making complicated procedures to the inexperienced nurse who has just started work
30.	Working in departments where the number of nurses is insufficient in terms of workload
31.	Working with the feeling of failure when the veins are not clearly visible and/or not felt, while blood is being drawn
32.	Working with limited decision-making powers
33.	Execution of procedures within the framework of negativities, such as incompatibilities, conflicts, etc. with student and trainee nurses
34.	Working with lack of time in terms of preliminary preparation and work planning
35.	Procedures resulting from lack of practice or encountering for the first time, inadequate intervention to critically ill patients
36.	Assisting the doctor within the procedures
37.	Increasing the workload of unaccompanied patients and completing the procedures depending on this workload
38.	To carry out the procedures within the scope of the problems caused by asking the questions that only the physicians can answer to the nurse
39.	To carry out the procedures within the negativities, such as inability to hear clearly in blood pressure measurement, and incompatibility of the patient's arm and cuff
40.	Working under undesirable conditions, such as problems, disorders, etc. in the functioning of medical devices
41.	Carrying out transactions within the framework of negativities, such as lack of spares of stationery tools and equipment, such as toner, paper, etc.
42.	Performing operations with psychology caused by situations, such as careful monitoring and interpretation of applications by patients/patient relatives
43.	Execution of transactions with the effects of directives and expectations of superiors
44.	Shift difficulties, lack of individual rights, and working in difficult working conditions
45.	Performing works that require intensive intervention and that have to complete the measurement and monitoring of vital signs in complex processes in limited and narrow times

## Abbreviations

HEART: Human error reduction and assessment technique
SWARA: Step-wise weight assessment ratio analysis
FSWARA: Fuzzy step-wise weight assessment ratio analysis
PCA: Principal component analysis
DSS: Decision support system
NTM-DSS: Nurse-task matching decision support system
HRA: Human reliability analysis
HEP: Human error probability
THERP: Technique for human error rate prediction
EPC: Error producing conditions
GTT: Generic task type
APOA: Assessed proportion of affect
EEG: Electroencephalography
MR: Magnetic resonance imaging
NASA: National Aeronautics and Space Administration
NASA-TLX: NASA Task Load Index
CFA: Confirmative factor analysis
MCDM: Multiple criteria decision-making
TD: Task difficulty
TP: Time pressure
P: Performance
MSE: Mental/sensory effort
PE: Physical effort
FL: Frustration level
SL: Stress level
F: Fatigue
AT: Activity type
PWT: Pairwise technique
